# IBC – Proteomics, 1–2 March 2000, Basel Hilton, Switzerland

**DOI:** 10.1002/cfg.80

**Published:** 2001-06

**Authors:** Philip John Masterson

**Affiliations:** KuDOS Pharmaceuticals Ltd 327 Cambridge Science Park Milton Road Cambridge CB4 0WG UK


**Comparative and Functional Genomics**
*Comp Funct Genom* 2001; **2**: 180–185.
**DOI**: 10.1002/cfg.80

**Meeting Review**

### IBC – Proteomics

### 1–2 March 2000, Basel Hilton, Switzerland

Philip John Masterson*
*KuDOS Pharmaceuticals Ltd, 327 Cambridge Science Park, Milton Road, Cambridge, CB4 0WG, UK*

**Correspondence to:*
*P. J. Masterson, KuDOS Pharmaceuticals Ltd, 327 Cambridge Science Park, Milton Road, Cambridge, CB4 0WG, UK.*
*E-mail: pmasterson@kudospharma.co.uk*

### Introduction

The massive increase in nucleotide sequence information available in public and private databases, coupled with advances in mass spectrometry (MS) and the associated search algorithms, have provided the basis for the emerging field of proteomics. The recent announcement of 35 000 or so genes in the human genome was on the low side of the number predicted. Yet, it re-affirms the view that cellular organisation is a complex system of protein complexes and networks of gene products. As **Walter Blackstock (Glaxo-Wellcome, UK)** pointed out 'We achieve our complexity not by sheer force of gene numbers, but by the infinitely subtle way in which gene products interact'. As a newcomer to the field it is clear that proteomics is now coming of age. At the end of the two day proteomics conference held at the Basel Hilton, Switzerland, my overriding impression was that momentum is gathering around the globe to catalogue and characterise the proteins encoded by the human genome, to compare variations in their expression levels under different conditions, study their interactions, and identify their functional roles. Proteomics on this scale requires new technologies and techniques and considerable effort is currently being devoted to the development of novel tools of the trade.

### 2DE and alternative separation techniques for proteome analysis

Whereas the conventional choice of two-dimensional electrophoresis (2DE) as the means for protein separation is open to discussion, there is no significant debate concerning the central role of MS as the analytical technique of choice. The first session contained a series of talks which highlighted the ability to interface mass spectrometry with a range of protein separation methodologies and established that sample preparation and presentation to the mass spectrometer play key roles in determining the successful outcome of any proteomics experiment.

The current and widely used method for protein separation relies on excising spots from gels, protein digestion, extracting the peptides produced and analysing the peptides by MS or tandem MS. An important limitation of the 2DE technology has been the lack of very sensitive procedures to detect those proteins that are present in very low abundance.

**Reid Townsend** described the 2DE based system employed at **Oxford Glycosciences (OGS), UK**, for high-throughput proteomic analysis of tissues and body fluids from normal and diseased individuals. High abundance proteins which may obscure the detection of proteins present in smaller amounts, that generally represent targets or biomarkers, are removed from body fluids using a rapid, automated process developed at OGS. A proprietary fluorescent dye, used in conjunction with a novel fluorescence scanner provides the basis of the OGS imaging techniques and analysis of tandem mass spectra is performed using the OGS database search algorithm, SEAL.

**Ed Hawkins (Amersham Pharmacia Biotech)** presented the fluorescence 2-D difference gel electrophoresis system (2D-DIGE), a technique whereby

Copyright © 2001 John Wiley & Sons, Ltd.

protein samples are labeled with different spectrally-resolvable fluorescent dyes so that they can be mixed together, co-separated and visualised on a single 2D gel for subsequent analysis. Running the mixed protein sample on the same gel clearly eliminates the problem of gel-to-gel variations. The fluorescent multiplexing of up to three proteins with different fluorescent dyes is compatible with mass spectrometry and offers sub-nanogram sensitivity for the analysis of 2D gels. Both these fluorescence detection procedures show a linear response to variation in protein concentration over five or six orders of magnitude and a broad dynamic range provides more accurate quantitative data than traditional 2D electrophoresis staining techniques.

General opinion seems to be that, at present, 2DE remains the method of choice for displaying proteins for two main reasons; first because it can be used to visualise a very large number of proteins simultaneously and, secondly, because it can be used in a differential display format, enabling the study of a biological system in its entirety rather than as a multitude of individual components. Limitations of the 2D analysis approach and potential solutions to these problems were addressed in a number of sessions. Methods of preliminary fractionation of samples were described to improve detection of low abundance proteins, as was the use of very narrow range immobilised pH gradient (IPG) strips for first dimension separation. The problem of detecting insoluble proteins is being addressed by study of new solubilisation solutions, including chaotropes and detergents, while to overcome the difficulties in detecting very large and very small proteins, people are looking to modify the gels themselves.

The discussion of 2DE above shows that there is room for improvement in the efficiency of proteomic analysis. This may be achieved either by advances in 2DE technology or through the development of new technologies. One such new methodology which moves away from a gel based system is known as Isotope Coded Affinity Tag (ICAT) peptide labelling [4]. The method is based on a newly synthesised class of chemical reagents (ICATs) used in combination with tandem mass spectrometry. This technique was discussed by **Tim Nadler from Applied Biosystems, USA**, who have optimised the protocol for derivatising and preparing samples with a modified labelling reagent. The ICAT reagent contains a biotin affinity tag and a thiol-specific reactive group, joined by a spacer domain, which is available in two forms; regular and isotopically heavy (includes eight deuterium atoms). Briefly, the reduced proteins of two different samples (control versus treated) are first labeled; one is derivatised with the ICAT reagent containing the stable heavy isotope and the other is labeled with the same reagent containing the lighter natural isotopes. The proteins are then mixed and proteolytically digested to produce peptide fragments. The tagged cysteine containing peptide fragments are isolated by avidin affinity chromatography, greatly reducing sample complexity and decreasing the amount of time required for MS/MS sequence analysis. An obvious advantage of this approach over 2DE is the potential for automation. Moreover, the technique provides accurate relative quantification of each peptide identified since the standard and sample peptide have the same sample ionization properties and only differ in mass. Limitations of the system include the fact that the proteins must first of all contain cysteine, which is true for approximately 80% of all proteins, and those cysteines must be flanked by appropriately spaced protease cleavage sites.

**Matthias Mann (MDS Proteomics, Denmark)** described a strategy of protein mixture analysis utilising data dependent mass spectrometers to generate as many peptides as possible from an enzymatically digested protein mixture. In a 'data-dependent analysis' the mass spectrometer measures the masses of eluting peptides and those that meet set signal threshold criteria are selected for fragmentation by the data system. Data-dependent experiments are usually conducted on peptide mixtures fractionated by reversed-phase liquid chromatography connected on line with the mass spectrometer. The procedure makes a complex mixture approach feasible and, again, the potential for automation is a key advantage. Moreover, the system has a very wide dynamic range and eliminates the problems of protein solubility associated with 2DE since the proteins are all proteolytically digested *en masse*.

**Randall Nelson (Intrinsic Bioprobes Inc, USA)** described the combination of surface plasmon resonance-biomolecular interaction analysis (SPR-BIA) and MALDI-TOF MS to facilitate the study of protein function and structure. The technology is focused around a sensor chip plated with gold **(BIACORE)**. The molecule under study is attached

Copyright © 2001 John Wiley & Sons, Ltd. 

to the surface of the sensor chip and sample solution is brought into contact with the surface. When a protein binds, the change in mass concentration close to the surface is measured in real time. The power of this approach is the simultaneous identification of unknown binding partners and the acquisition of data regarding the specificity, affinity and kinetics of the interaction. Recently SPR-BIA has been combined with tandem MS in order to sequence proteins bound to the sensor surface chip. The proteins bound to the sensor chip are enzymatically digested on the chip by delivering proteolytic enzyme to the flow cell via a microfluidics system. To minimise the risk of sample loss the resulting peptide mixture is then trapped in a capillary precolumn by an on line recovery technique for subsequent tandem MS analysis. The introduction of this technique vastly improves the accuracy of protein identification in proteomic applications.

### Functional Proteomics

Today, the term proteomics encompasses much of the functional analysis of gene products or 'functional genomics'. In the short term, the goal of functional genomics is to assign some element of function to each of the genes in an organism and to do this with high throughput. Long-term efforts will focus on elucidating the organisation and control of genetic pathways that come together to make up the physiology of an organism. Knowledge of protein-protein interactions helps place novel proteins in their functional context. **Walter Blackstock (Glaxo-Wellcome, UK)** suggested that Interaction or Cell Map proteomics 'will play an important role in teasing out the protein complexes and networks of gene products by which we achieve our cellular complexity'.

In the majority of cases functional proteomic approaches involve the isolation of a subset of proteins from a given starting material through a variety of affinity-based methods (GST-fusion proteins, antibodies, peptides, DNA, RNA or a small molecule binding specifically to a cellular target). Additionally, the enrichment afforded by affinity purification facilitates the detection of low copy number proteins and, monitoring a reduced subset of proteins makes differences easier to detect in differential display experiments. In his keynote address, **Matthias Mann** described as an example the purification of the human spliceosome using biotinylated RNA as the bait on which the complex assembled [5]. Its protein components were then displayed by 2DE and 19 new components identified from a single gel. He went on to describe, using the yeast nuclear pore complex Nup85p as an example, how spatial organisation of multi-protein complexes can be established through carefully controlled cross-linking conditions to achieve coupling of adjacent proteins. Components of specific organelles have also begun to be analysed. **Dr. Mann** described how work in collaboration with Angus Lamond (Dundee) had so far identified more than 200 nucleolar proteins of which 23% are novel. **Walter Blackstock** outlined the tandem affinity purification strategy (TAP) developed by Rigaut *et al.* [8] which has been shown to improve complex recovery and reduce non-specific protein binding. The method involves double-tagging a known protein component of a complex of interest and introducing this construct into the host cell. The native portion of the tagged protein then interacts with cellular proteins, the complex is recovered through two affinity column steps and the components are identified by MS and database searching.

**Timothy Haystead (Duke University, USA and Serenex, USA)** outlined a functional proteomic approach to identify bioactive ligands and their physiological targets *en masse*. The technology utilises naturally occurring bioactive molecules, such as ATP, required *in vivo* to enable normal cellular processes to occur. Whole tissue/cell extract is passed through a 'proteome mine', in the case of an ATP mine this consists of prepacked ATP sepharose column cassettes. Following removal of non-specifically bound proteins the columns are successively washed with libraries of synthetic and semi-natural product derived purine nucleotide analogs and fractions collected and screened for the presence of protein. The elution process is repeated with additional library components (up to 10 structurally non-related components at a time). Each proteome mine unit containing a 12 column cassette can screen 1200 compounds per day if each elution represents 10 library components. Fractions containing protein are characterised by SDS-PAGE and the proteins identified by mixed peptide sequencing or MS. If the protein is deemed relevant then the mining process will immediately generate a lead compound and its physiological target. A repertoire of resins is currently being developed as

Copyright © 2001 John Wiley & Sons, Ltd. 

mines and include all purine and pyrimidine based nucleotides (for DNA and RNA binding proteins), Calmodulin (for $Ca^{2+}$ regulated proteins), and microcystin (for protein phosphatases).

A number of biotechnology companies are gearing up towards high-throughout methods for identifying and characterising all of the proteins, protein domains and protein interactions in a cell using a functional proteomics approach. **MDS Proteomics (Denmark)** has created an integrated functional proteomics and drug discovery platform to reveal cellular pathways, identify novel targets, and discover new therapeutic drugs and diagnostic products. **Ron Hendrickson** outlined MDSPs platform strategy of identifying critical interactions and targets using multiple 'baits' to isolate protein complexes (PathMap™), the use of LeadFinder™ to prioritise and quantitate hits and generate structural information for drug discovery, and the storing and interpretation of information for target selection using the Biomolecular Interaction Network Database (BIND). The BIND database (**http://bioinfo.mshri.on.ca/cgi-bin/bind/dataman**) is a publicly available bioinformatics database that provides a means of efficiently representing complex cellular pathway information *in silico*. BIND will allow researchers worldwide to submit and review results of research defined in three main data types: interactions, molecular complexes, and pathways.

**Dr. Donny Strosberg (Hybrigenics)** described how the company's basic technologies are derived from protein-protein interaction analyses using cell-based methods such as the two-hybrid in yeast, in bacteria techniques (bacterial two-hybrid), or biophysical analysis based on BRET (Bioluminescence Resonance Energy Transfer) technology. Using genomic DNA from microbial pathogens or cDNA from normal or diseased tissues, Hybrigenics strategy is to identify interacting proteins, reconstitute functional signaling or metabolic pathways and finally identify modulators of these interactions which may constitute lead compounds. Comprehensive Protein Interaction Maps (PIMs) representing the interaction network of the proteins of an organism or between the proteins of two different organisms are being created. Each interaction is evaluated and given a reliability score. Hybrigenics' approach of two-hybrid screens reveals Selected Interacting Domains (SIDs) which comprise the common part of all overlapping prey fragments isolated in a particular screen and contains the protein-protein interaction domain. Hybrigenics then uses SIDs and libraries of small molecules to identify lead compounds capable of modulating interactions. The strategy has recently been used to build up a large-scale protein-protein interaction map of the human gastric pathogen *Helicobacter pylori* [7].

### Proteo-Informatics

High-throughput biotechnology calls for strong bioinformatics tools in the form of powerful biological data management systems and specific algorithms for processing these data. Bioinformatics pervades each step of the high-throughput proteomic analyses outlined above. In all talks in this session, speakers urged that this sort of management of complex biological data is essential, now, before the information accumulated becomes overbearing.

The analysis of gel images to extract pertinent biological information still represents a major bottleneck in large-scale proteomics projects. **Sonja Voordijk (Geneva Bioinformatics)** outlined the challenges for 2D gel analysis software with examples of their product, Melanie 4, in action. The new generation of software systems will not only have to ensure high quality spot quantitation and accurate, robust spot matching (which are the crucial steps in 2DE gel analysis), they will also have to enable the handling of large volumes of 2DE data in an automated way. In addition, they will need to contain advanced statistical and classification functions and allow unlimited annotation capabilities with the possibility to link and associate gel objects to external equipment or data of any format located locally or on the internet. Other image analysis packages of note such as ImageMaster **(Amersham-Pharmacia Biotech)** and Phoretix **(Nonlinear Dynamics)** were presented in tutorial sessions.

The data management systems may be complemented by information databases such as those compiled at **Proteome, Inc., USA** (**http://www.proteome.com/databases/index.html**). **James I. Garrels**, talked of Proteome's provision of knowledge resources combining information on the proteins and genes of human, mouse, rat and several other model organisms. Proteome's curators review scientific literature to produce the proteome databases, which contain indexed biological information on proteins and can be used for annotating and interpreting experimental

Copyright © 2001 John Wiley & Sons, Ltd. 

results from proteomics studies. Algorithms have also been generated which enable proteins to be grouped according to their sequences with the assumption that such clustering of proteins would lead to functional grouping. In this way networks of functional linkages between proteins in cells can be identified.

### Protein Arrays

Ordered arrays of peptides and proteins provide the basis of an alternative strategy to 2DE for parallel protein analysis. A range of chip-based and array-based technologies are emerging for the identification and characterisation of individual proteins and for the profiling of protein expression in cells. At **Ciphergen Biosystems, USA**, for instance, researchers have developed a technique called ProteinChip in which a crude biological sample is placed on an array capable of capturing a subset of proteins from the sample. The array surface can be a chemical surface with broad specificity so that it catches whole classes of proteins. Or it can be extremely specific, such as an antibody-, enzyme-, or receptor-based surface that is highly selective for a few proteins. After the capture step, the ProteinChip array is washed and the proteins retained on the surface are analysed by MS.

Proteome analysis of the simplest self-replicating organism, *Mycoplasma genitalium* revealed an error rate of at least 8% in the annotations for 340 genes [9]. **Ian Humphery-Smith (University of Utrecht and Glaucus Proteomics)** suggested that the experience gained from such studies had taught many valuable lessons and that efforts to tackle the complexity of human beings could centre around non-traditional approaches such as protein, peptide and antibody arrays. A strategy of emulating the diversity encoded by the genome through parallel generation and screening of antibodies was discussed. The key steps in large-scale antibody production including the functionality of screening engines capable of processing 50 000 000 ELISA-equivalents per day per robot were outlined.

Recombinant antibodies are becoming increasingly important in the field of proteomics. Recent advances include the development of large phage antibody libraries that contain high affinity binders to almost any target, and new methods for high throughput selection of antibody-antigen interaction. Phage display is a method where bacteriophage particles are made to express either a peptide or protein of interest fused to a capsid or coat protein. **Lucy Holt (MRC LMB, UK)** described a novel technique for high-throughput screening of recombinant antibodies, based on the creation of antibody arrays. The method uses robotic picking and high density gridding of bacteria containing antibody genes followed by filter based ELISA screening to identify clones that express antibody fragments which bind the antigen(s) under test. By eliminating the need for liquid handling, up to 18 342 different antibody clones can be screened at a time and, because the clones are arrayed from master stocks, the same antibodies can be multiply spotted and screened simultaneously against up to 15 different antigens. Results so far suggest that the antibody arrays can be used to identify differentially expressed proteins [3].

### Applications of proteomic analyses

Expression proteome analysis or Differential proteomics compares the expression profile of 2DE separated proteins from an arbitrary reference state of a cell, tissue, or organism, to the profile of a non-standard condition, such as a diseased state or after the addition of a toxin to the system. **Julio Celis (Danish Centre for Human Genome Research, Denmark)** highlighted the potential of Expression proteomics in the area of molecular medicine to identify protein markers or protein patterns which change in association with disease progression. Such protein biomarkers can be used to identify disease prior to the appearance of physical symptoms and distinguish between drug efficacy and toxic side effects, as well as identify new disease pathways and drug targets. In one ongoing project, Celis and colleagues have used proteome expression profiles to reveal and identify proteins that are differentially expressed in pure squamous cell carcinomas (SCC's) and normal urothelium of bladder tumours and used them as fingerprints to subclassify histopathological types and as a starting point for searching for protein markers. Specific antibodies against the identified differentially expressed proteins have been used to immunostain serial cryostat sections of biopsies obtained from SCC patients that have undergone removal of the bladder due to invasive disease. So far these studies have revealed markers for transitional cell carcino-

Copyright © 2001 John Wiley & Sons, Ltd. 

mas (TCC) [2], a marker in the urine of patients bearing SCCs [6], and have led to the development of a novel strategy for identifying premalignant squamous lesions [1]. In the course of his talk the difficulties of using human tissue to study proteins were described and included preserving the state of the proteins in the tissue sample and the heterogeneity of the tissue itself. A piece of tissue from an organ or any part of the body contains numerous different interacting cell sub-populations of which only a tiny percentage of the total cells are diseased. Consequently, there is a challenge to separate out the subset of cells that are of interest either by Laser Capture Microdissection or by enzymatic digestion of the cells followed by a cell fractionation process.

Another notable study described efforts to elucidate the molecular events leading to cardiac dysfunction and was presented by **John Weekes (Imperial College of Science, Technology and Medicine, UK)**. Using a differential proteomic analysis, Dr. Weekes and colleagues have demonstrated that upregulation of the ubiquitin-proteasome system of proteolysis occurs in dilated cardiomyopathy (DCM). This leads to hyper-ubiquitination of a number of cardiac proteins. Ubiquitinated proteins were purified using S5a-Sepharose, affinity chromatography and separated using 2DE. All the identified ubiquitinylated proteins showed reduced expression in DCM hearts indicating that inappropriate proteolysis of these proteins by the proteasome may be a critical factor leading to loss of normal cellular activity in the DCM heart and ultimately to heart failure.

### Concluding Remarks

Harnessing the available data in the post-genome era is the major challenge facing many biological scientists. Five years ago, when Mark Wilkins coined the term proteome, few researchers were even contemplating a wholesale protein discovery effort on the scale of the human genome project. Today, it's a very different picture and the field's aspirations have gained legitimacy. As is evident from proteomic meetings such as this one, researchers are turning increasingly to the task of converting the completed human DNA sequence into information that will potentially improve, and perhaps revolutionise human medicine and health care. Advances in proteomic technologies which lead to faster sample throughput and increased sensitivity for the detection of individual proteins will facilitate the realisation of this objective.

### References

1. Celis JE, Celis P, Ostergaard M, *et al.* 1999. Proteomics and immunohistochemistry define some of the steps involved in the squamous differentiation of the bladder transitional epithelium: A novel strategy for identifying metaplastic lesions. *Cancer Res* **59**: 3003–3009.
2. Celis JE, Ostergaard M, Basse B, *et al.* 1996. Loss of adipocyte-type fatty acid binding protein and other protein biomarkers is associated with progression of human bladder transitional cell carcinomas. *Cancer Res* **56**: 4782–4790.
3. de Wildt RMT, Mundy CR, Gorick BD, Tomlinson IM. 2000. Antibody arrays for high-throughput screening of antibody-antigen interactions. *Nat Biotechnol* **18**: 989–994.
4. Gygi SP, Rist B, Gerber SA, Turecek F, Gelb MH, Aebersold R. 1999. Quantitative analysis of complex protein mixtures using isotope-coded affinity tags. *Nat Biotechnol* **17**: 994–999.
5. Neubauer G, King A, Rappsilber J, *et al.* 1998. Mass spectrometry and EST-database searching allows characterization of the multi-protein spliceosome complex. *Nat Genet* **20**: 46–50.
6. Ostergaard M, Wolf H, Orntoft T, Celis JE. 1999. Psoriasin (S100A7): A putative urinary marker for the follow-up of patients with bladder squamous cell carcinomas. *Electrophoresis* **20**: 349–354.
7. Rain JC, Selig L, De Reuse H, *et al.* 2001. The protein-protein interaction map of *Helicobacter pylori*. *Nature* **409**: 211–215.
8. Rigaut G, Shevchenko A, Rutz B, Wilm M, Mann M, Seraphin B. 1999. A generic protein purification method for protein complex characterization and proteome exploration. *Nat Biotechnol* **17**: 1030–1032.
9. Wasinger VC, Pollack JD, Humphery-Smith I. 2000. The proteome of *Mycoplasma genitalium*—Chaps-soluble component. *Eur J Biochem* **267**: 1571–1582.

The Meeting Reviews of *Comparative and Functional Genomics* aim to present a commentary on the topical issues in genomics studies presented at a conference. These reviews are invited and each represents a personal critical analysis of the current reports and aim at providing implications for future genomics studies.

Copyright © 2001 John Wiley & Sons, Ltd. 
